# Supplementary figures and images for: Global, regional, and national burden of near vision loss in children and adolescents under 20 years from 1990–2021 and prediction to 2060: A cross-sectional study based on the global burden of disease study 2021
Source: PLoS One. 2025 Jul 8;20(7):e0327000. doi: 10.1371/journal.pone.0327000 (PMC12237012; doi:10.1371/journal.pone.0327000)

# Global

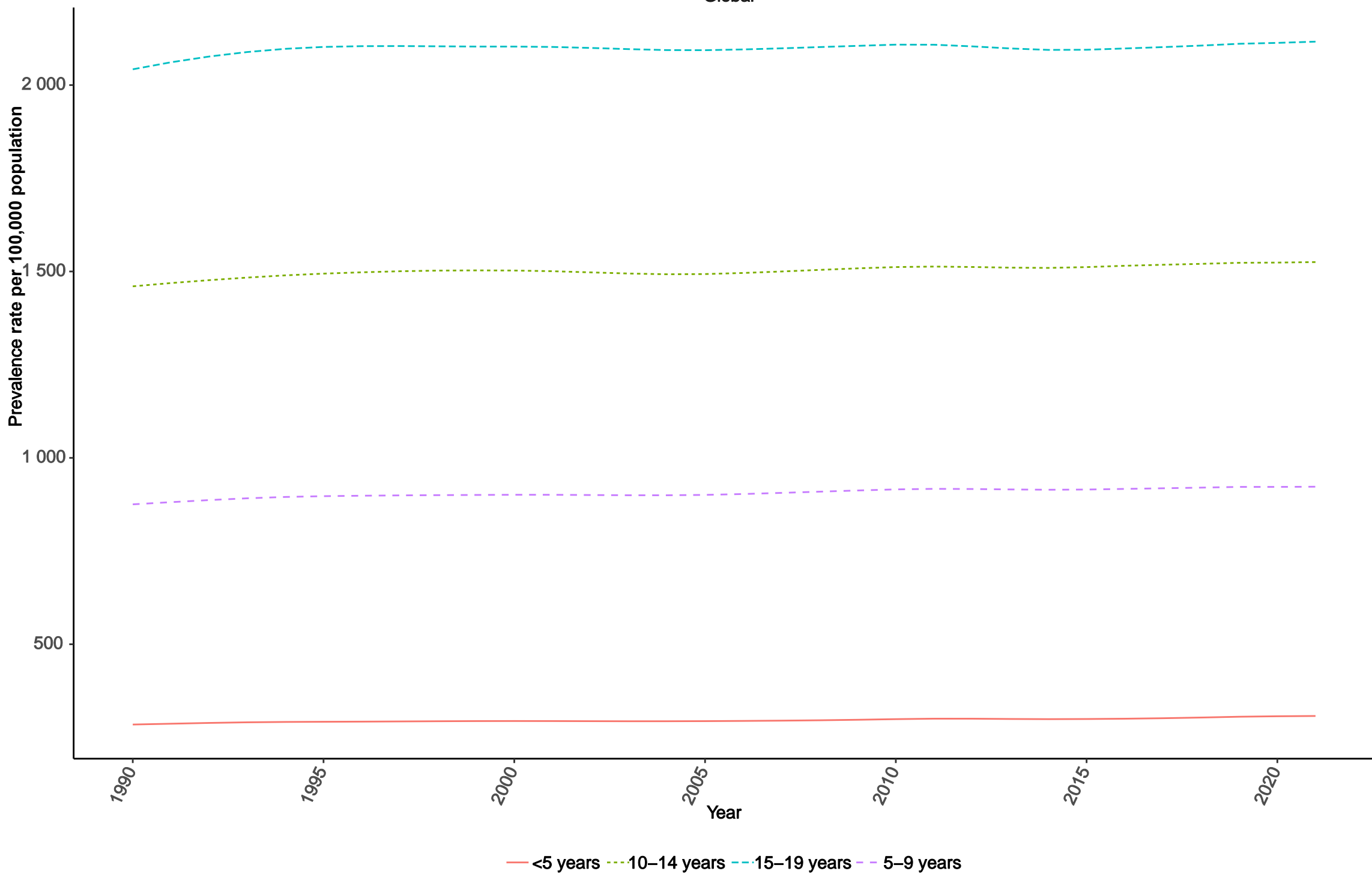

Supplement: S1 Fig — (PDF) [file pone.0327000.s001.pdf]
